# Using High-density Grid Technology to Map Intramural Left Ventricular Summit Premature Ventricular Complexes with Associated Cardiomyopathy

**DOI:** 10.19102/icrm.2021.120122S

**Published:** 2021-01-15

**Authors:** Daniel Alyesh, William Choe, Austin Davies, Sri Sundaram

**Affiliations:** ^1^South Denver Cardiology Associates, Littleton, CO, USA; ^2^Abbott, Chicago, IL, USA

**Keywords:** Cardiomyopathy, intramural PVC, LV summit

A 69-year-old woman with hyperlipidemia and ocular cicatricial pemphigoid presented with frequent (burden: 47%) symptomatic (ie, palpitations) premature ventricular complexes (PVCs) with an outflow tract morphology and associated cardiomyopathy [left ventricular (LV) ejection fraction: 40%]. She underwent a negative ischemic workup and was started on guidelines-directed medical therapy without improvement in her LV function.

She underwent an initial PVC ablation using activation mapping with an ablation catheter and radiofrequency (RF) energy was delivered from the right ventricular outflow tract (RVOT), LV outflow tract (LVOT), and right–left coronary commissure. Unfortunately, the PVC recurred three months later and her cardiomyopathy continued.

She presented for a redo ablation with the Advisor™ HD Grid Mapping Catheter, Sensor Enabled™. Initial activation mapping in the RVOT revealed late timing as was the case in the coronary cusps. Meanwhile, activation mapping with the Advisor™ HD Grid catheter in the LVOT revealed an early fractionated signal just below the left coronary cusp **(Figure 1)**. Activation mapping was then performed in the coronary sinus at the level of the anterior interventricular vein with an ablation catheter. Early far-field timing was also noted in this location.

A coronary angiogram revealed the ablation catheter to be at a safe distance from the left anterior descending coronary artery. Ablation was performed in this region at 20 W with suppression of the PVC achieved after 15 seconds, but it returned after 30 seconds. Attention was then turned to the early signal in the LVOT identified with the Advisor™ HD Grid catheter. Ablation at this site with 35 W eliminated the PVC within five seconds **(Video 1)**.

At five months postablation, the patient remained without any further PVCs and with normalization of her LV function. Guidelines-directed medical therapy is gradually being weaned. This case demonstrates the greater value of high-resolution signal detection with the Advisor™ HD Grid catheter in the case of intramural PVCs relative to traditional ablator-based mapping.

## Figures and Tables

**Figure 1:**
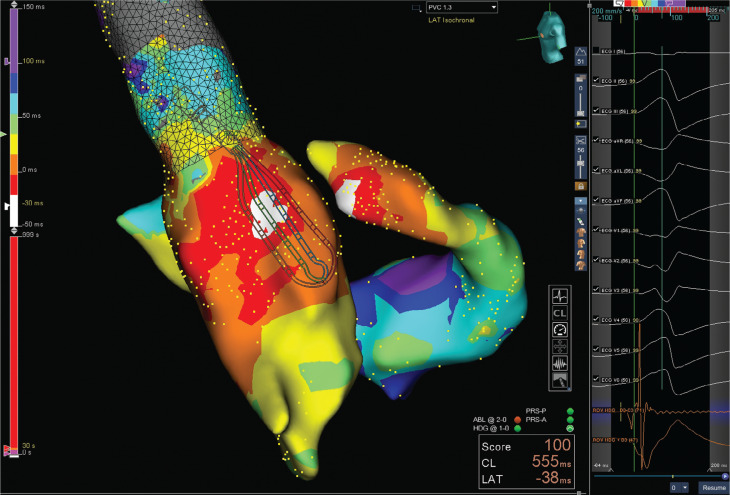
High-density mapping performed with the Advisor™ HD Grid catheter in the LVOT just across the aortic valve identified an early bipolar signal with a corresponding good unipolar signal.

**Video 1.** Application of RF energy at the early site identified with the Advisor™ HD Grid in the LVOT eliminated the PVC within five seconds.

